# Self-Incompatibility Assessment of Some Italian Olive Genotypes (*Olea europaea* L.) and Cross-Derived Seedling Selection by SSR Markers on Seed Endosperms

**DOI:** 10.3389/fpls.2019.00451

**Published:** 2019-04-12

**Authors:** Cinzia Montemurro, Giovanni Dambruoso, Giovanna Bottalico, Wilma Sabetta

**Affiliations:** ^1^Department of Soil, Plants and Food Sciences, Faculty of Agricultural Science, University of Bari “Aldo Moro,”, Bari, Italy; ^2^SINAGRI S.r.l. – Spin off University of Bari, Bari, Italy

**Keywords:** olive, self-compatibility, *in vitro* embryo culture, endosperm, SSRs analysis

## Abstract

The morphology of olive flowers allows either self- or cross-pollination that could partially explain the existence of both reproductive features in this species. However, a high degree of self-incompatibility is reported for many olive genotypes, that could be an important reproductive barrier influencing olive yield. Due to the strong environmental influence, results of compatibility tests are often contradictory, making cultivar classification quite imprecise. In this study, the self-incompatibility value has been determined for four olive genotypes (Bella di Spagna, Coratina, Leccino, and Ogliarola barese) widespread in the Mediterranean basin. Moreover, the incompatibility relationships of cultivar Coratina with some suitable pollinizers (Leccino, Oliastro, and Picholine) have been studied in controlled crosses: the *in vitro* germination potential of progenies has been evaluated and the selection of cross-derived embryos has been indirectly performed by the molecular characterization of the corresponding endosperm. The results increase knowledge on factors affecting self-compatibility in olive. Moreover, they provide useful information to farmers about the most effective cultivars for the set-up of new olive grove or for graft planning. Finally, they provide a new strategy and procedure based on endosperm analysis by SSRs for an accurate, fast, and relatively cheap screening of embryos/seedlings.

## Introduction

Like many hermaphrodite plants, olive (*Olea europaea* L.) is characterized by copious flowering followed by relative poor fruit set (Stephenson, 1981; Burd, 1998). This could be due to different reasons, such as a high incidence of ovary abortion (female sterility) (Reale et al., 2006; Rosati et al., 2011), or to reproductive barriers, including pollen sterility (Besnard et al., 2000) and self-incompatibility (Bateman, 1952). Self-incompatibility is certainly the most important reproductive barrier influencing olive yield. It evolved from self-compatibility and is a natural mechanism promoting outbreeding, genetic variability, and consequently evolutionary diversification in allogamous plants (Igic et al., 2008; Locato et al., 2016).

The morphology of olive flowers allows either self- or cross-pollination, thus partially explaining the existence of both reproductive features. However, despite the first idea that classifies olive as a totally or partially self-compatible species (Fernàndez-Bolanòs and Frìas, 1969), several studies have confirmed the presence of a high degree of self-incompatibility (Mookerjee et al., 2005; Diaz et al., 2006; Farinelli et al., 2008; Serrano et al., 2008). The debate about olive mating system has recently taken on controversial tones and thereby needs further clarification. Indeed, there is debate about a classical sporophytic self-incompatibility (SSI) system (Breton and Bervillé, 2012; Breton et al., 2014, 2016; Farinelli et al., 2015) and an unusual, homomorphic, diallelic self-incompatibility (DSI) system based on reciprocal stigma tests among olive genotypes (Saumitou-Laprade et al., 2017a). The main causes of incongruent results of these studies have been largely discussed by the same authors (Saumitou-Laprade et al., 2017b; Farinelli et al., 2018). Recently, the whole flower transcriptome analysis of three olive cultivars (Leccino, Frantoio, and Dolce Agogia) allowed to identify key genes involved in ovary abortion, pollen–pistil interactions, and self-incompatibility and thereby to shed some light to these biological processes (Alagna et al., 2016). Finally, several authors attribute the contradictory results of olive compatibility tests to location, year, experimental methodology (Bartolini and Guerriero, 1995; Cuevas and Polito, 1997; Cuevas et al., 2001; Lavee et al., 2002; Camposeo et al., 2012). It is of common acceptance the strong influence of environmental and climatic conditions and of type of orchards on self-incompatibility reaction and fruit set, in particular during the critical period of blooming and pollination stage (Ghrisi et al., 1999; Lavee et al., 2002). Consequently, cultivar classification as self-compatible or self-incompatible is quite confused so far.

The geographical position in the Mediterranean area and its temperate clime have made Southern Italy an optimal territory for olive cultivation (Bartolini and Petruccelli, 2002; Cimato and Attilio, 2011). Both the presence of ancient and wild trees and the diffusion of modern cultivars are a testimony of the great importance that this species has assumed over the time for the economy, culture, and local traditions of Southern Italian regions. Among the Italian olive germplasm, the cultivars Coratina, Ogliarola barese, Leccino, Bella di Spagna, Picholine, and Oliastro are particularly widespread for their features. In particular, the Coratina oil is rich of unsaturated fatty acids and phenolic compounds, that makes it extremely beneficial for human health (Servili et al., 2004; Alagna et al., 2009). On the other hand, the bitterness and pungent flavor characteristic of this oil, requires qualified and conscious consumers; since the market promotes sweeter and balanced oils, the Coratina oil is mostly used in blend (Aparicio and Luna, 2002; Rotondi et al., 2013 ^1^). However, the economic importance of Coratina, together with cultivars Leccino and Ogliarola barese, is also highlighted by their use in several Apulian PDO foodstuff. Moreover, each of these cultivars has interesting traits, such as high productivity, tolerance/resistance to some of the most important olive pathogens, rusticity, and adaptability to various environmental conditions (Sofo et al., 2004; Sanzani et al., 2012; Giampetruzzi et al., 2016), thus validating their use in olive breeding programs. The self-compatibility or -incompatibility of these cultivars is not well known yet, despite the presence of some studies (Farinelli et al., 2004, 2006, 2015; Al-Kasasbeh et al., 2005; Guerin and Sedgley, 2007; Seifi, 2009; Camposeo et al., 2012; Fayek et al., 2014).

The nature of olive seeds, covered by a stony endocarp and characterized by embryo dormancy, makes the germination period extremely long in this species. To overcome such problems, the *in vitro* culture of isolated embryos has been successfully employed in olive as well as in many other fruit species (Rugini, 1984, 1986; Ramming, 1990; Germanà et al., 2009; Abdallatif et al., 2015), providing a method to obtain plantlets within a reasonable time suitable for further manipulations in genetic improvement programs. In addition, the partial self-compatibility nature of olive varieties raises the question of ascertaining accurately the origins of drupes, if by cross- or self-pollination. The employment of molecular markers for this purpose has represented a solution to the drawback of morphological traits, traditionally used in the past, by their application to DNA extracted from plants under study, never to DNA from the corresponding seed endosperms. In particular, the use of microsatellite markers (SSR) is notoriously simply, relatively cheap, reliable, and specie-specific (Sefc et al., 2000; Carriero et al., 2002; Cipriani et al., 2002). In olive, they have been used for different purposes, such as cultivar identification (Alba et al., 2009), assessment of genetic diversity (Albertini et al., 2011), evaluation of relationships among cultivated and wild olives (Boucheffa et al., 2017), designation of geographic origin (Sarri et al., 2006; Montemurro et al., 2015), genetic mapping (De la Rosa et al., 2003), construction of core collections (Haouane et al., 2011), oil traceability (Pasqualone et al., 2013, 2016; Sabetta et al., 2017), paternity test (De La Rosa et al., 2004; Dìaz et al., 2007; Arbeiter Baruca et al., 2014), and evaluation of polyploidy level (Besnard and Baali-Cherif, 2009).

Among more than 250 genera in which self-incompatibility occurs, including most of fruit species, in recent years, olive has been considered a case-of-study for the investigation of the mechanisms underlying self-incompatibility. This highlights the importance of studies assessing self-compatibility among cultivars, which consequently can lead to the most successful choice of genotypes to set-up new and improved olive groves or to plan olive grafts. The objectives of our research were multiple: (1) to determine the self-compatibility values of four Italian olive cultivars (Bella di Spagna, Coratina, Leccino, and Ogliarola barese) by the evaluation of number of flowers per inflorescence (NFI), percentages of perfect flowers and fruit sets in two different locations of Southern Italy and under two different types of pollination (self and free); (2) to study the compatibility relationships of one of these cultivars, i.e., Coratina, with some suitable pollinizers (Leccino, Oliastro, and Picholine). This second goal was reached by the evaluation of the *in vitro* germination potential of progenies derived by controlled crosses, using mature embryos as explants, and their molecular characterization; (3) to establish and apply the SSR analysis, for the first time, to DNA extracted from seed endosperms (not from leaves), for an accurate and fast indirect embryo selection that allows to save time, money, and laboriousness.

## Materials and Methods

### Plant Material

Four Italian olive cultivars, extensively spread in Southern Italy, have been analyzed: Coratina, Leccino, and Ogliarola barese, mainly cultivated for oil production, and Bella di Spagna, as source of table olives. The experiments were carried out in 2014 and 2015, in two plantation orchards, located in different districts of Apulia region: the Olive Pre-Multiplication Centre and the Department of Soil, Plants and Food Sciences (Di.S.S.P.A) olive collection. The olive collection at the Olive Pre-Multiplication Centre of Palagiano (Ionic site) is located in the province of Taranto, on the Apulian West coast that is characterized by mild temperature and absence of cold wind ^2^. The orchard harbors 19-year old trees belonging to 22 olive cultivars placed in rows with cultivation distance of 4.5 m × 4.5 m; plants are drop-irrigated every 7 days and fertilized twice per year. The Di.S.S.P.A olive collection of University of Bari (Adriatic site) is located at the experimental field “Martucci” in Valenzano, close to Bari, on the Apulian East coast, that is characterized by windy and dried springs (www.meteoam.it). The mixed orchard harbors eight olive cultivars and other fruit tree species, such as apricot, cherry, fig, plum, pear, walnut, and almond, with a cultivation distance of 7.0 m × 7.0 m. The 24-year-old olive trees are weekly watered and pruned every 2 years. Fertilization and herbicidal treatments are applied twice per year.

### Flower Morphological Sterility and Pollination Test

For each cultivar, five plants with similar canopy size, flowering time uniformity, and good vegetative status were chosen to perform morphological sterility and self-compatibility tests. Sixty apical branches per plant were selected and the total number of inflorescences was taken. For a representative sample of 30 inflorescences per plant, the average NFI and the numbers of perfect flowers per inflorescences [hermaphroditic flowers (HFs)] have been recorded. The total number of flowers was obtained multiplying the number of flowers/inflorescence for the number of inflorescences/branch. When flower buds were at the balloon stage (white, swollen, and near to open) (Fayek et al., 2014), 50 out of 60 branches were isolated with TNT bags (made of a synthetic polypropylene fiber, water, and pollen-proof) for self-pollination test, while flowers from 10 branches for each tree were left to pollinate under natural conditions. Bags were removed four weeks later. The fruit set per branch was evaluated at 40, 90, and 120 days after flowering (daf) and fruits from self-pollination and open pollination were collected in November.

### Controlled Cross Pollination

One out of the four analyzed cultivars, i.e., Coratina, was chosen for cross pollination under controlled conditions. Plants involved in these experiments exclusively belonged to the olive collection located in Palagiano (TA). The cv Coratina was used as female parent in three crosses, respectively, with cultivars Leccino, Oliastro, and Picholine. Several plants as female and male parents with flowering contemporaneity were selected for each cross. The apical branches of male parents with flowers at the balloon stage were double-bagged, using an inner paper bag and an outer TNT bag. The pollination procedure was repeated twice, every 7 days, in order to optimize the pollen collection. In the same period, some branches of the female parent were also isolated with TNT bag to avoid uncontrolled contamination and used as self-pollination control. At the beginning of anthesis, the bags containing pollens were took away from male branches and immediately used to replace bags on the host isolated branches. These branches were further coated with larger TNT bags. Periodically, the bags were slightly shaken manually to promote pollination of all flowers. In November, fruits derived from self- and cross-pollinated branches were collected and pitted; the endocarps were treated with 1 M NaOH for 30 min to remove mesocarp residues, then washed, air-dried, and stored at 4°C for 30 days to overcome dormancy.

To evaluate the degree of self-fertility of the studied cultivars, the index of self-incompatibility (ISI) was calculated according to Androulakis and Loupassaki (1990): the fruit set ratio in self- and cross-pollination conditions classified the cultivars as completely self-incompatible (0), mostly self-incompatible (0–0.15), partially self-incompatible (0.15–3), and self-compatible (0.3–1).

### Embryo Isolation and *in vitro* Culture

The woody endocarps were broken by a vise and the extracted seeds were soaked in water for 48–72 h; then, they were surface-sterilized with 70% (v/v) ethanol (Sigma-Aldrich, St. Louis, MO, United States) for 30 s, followed by 15 min immersion in a solution of 0.05% (v/v) HgCl_2_ (Fluka-Sigma-Aldrich) and 0.04% Tween 20 (Sigma-Aldrich, St. Louis, MO, United States) with gentle shaking, and finally rinsed with three changes of sterile distilled water for 10 min each. Sterilized seeds were longitudinally and transversely cut and embryos were carefully removed under sterile conditions and individually placed in tubes containing 25 mL of 4.3 g/L MS medium (Murashige and Skoog, 1962), 10.0 g/L sucrose (Sigma-Aldrich, St. Louis, MO, United States), 500 mg/L calcium nitrate tetra-hydrate (Duchefa Biochemie, RV Haarlem, Netherlands), and 3.5 g/L Phytagel (Sigma-Aldrich, St. Louis, MO, United States), pH 5.8. Each surrounding endosperm was collected, marked with the same label of the corresponding isolated embryo, and stored at -20°C for molecular analysis. The culture tubes were closed with plastic caps and placed in a growth chamber at 20°C with a 16 h photoperiod. The *in vitro* embryo development was weekly monitored; when seedlings reached the stage of four-well developed leaves and had 2 cm long secondary roots, they were moved to pots containing sterilized peat-moss, sand and soil in the ratio of 1:1:1, and kept in the growth chamber for acclimation. After some weeks, plantlets with a good foliar and rooting apparatus were moved to the greenhouse.

### DNA Extraction From Endosperms and SSR Analysis

Genomic DNA was extracted from endosperms of the progenies derived by each cross and by host self-pollination. The Gene Elute Plant commercial kit (Sigma-Aldrich, St. Louis, MO, United States) was used. DNA quality and concentration were assessed by means of 0.8% (w/v) agarose gel electrophoresis and NanoDrop^TM^ 1000 Spectrophotometer (Thermo Scientific, United States), respectively. DNA was normalized to a standard concentration of 50 ng/μL by adding 0.1 × TE buffer (10 mM Tris-HCl pH 8.0 and 1 mM EDTA).

Two SSR markers, UDO-43 (Cipriani et al., 2002) and EMO-L (De la Rosa et al., 2002), clearly polymorphic between parents of each cross and with proven amplification efficiency, reproducibility, quality of scoring, information content, and discrimination capacity (Alba et al., 2009; Baldoni et al., 2009), were selected to analyze the respective progeny. Amplification reactions were performed in a programmable thermal cycler (Bio-Rad Laboratories, Hercules, CA, United States), in a final volume of 12.5 μL containing 30 ng of DNA, 1X PCR buffer, 0.25 mM dNTP, 2.5 μM each primer (Life Technologies, Carlsbad, CA, United States), and 1 U Euroclone DNA polymerase (Euroclone-Celbio, Pero, Italy). The forward primer of each microsatellite was labeled with FAM or NED fluorophores. The amplification conditions were: 5 min at 94°C; 1 min at 94°C, 1 min at the appropriate annealing temperature, and 2 min at 72°C per 35 cycles; final elongation at 72°C for 30 min; 2 μL of each PCR product were added to a mixture containing 14.5 μL formamide and 0.5 μL of the GeneScan^TM^ 600 LIZ^®^ Standard Size (Applied Biosystems, Warrington, United Kingdom). Samples were denatured at 94°C for 5 min, and then separated by capillary electrophoresis on an ABI PRISM 3130 Avant Genetic Analyzer (Applied Biosystems, Warrington, United Kingdom). The obtained electropherograms were acquired and analyzed by GeneMapper v.5 software (Applied Biosystems, Warrington, United Kingdom).

### Experimental Design and Statistical Analysis

This study was designed according to a randomized complete design. Data related to NFI, number of perfect flowers (HF) and percentage of fruit set were subjected to variance analysis (ANOVA) according to Duncan’s test (Duncan, 1955; Lison, 1961) by the use of MSTAT-C statistical package software (Nissen, 1983). The FACTOR function was applied to analyze the fruit set percentage, evaluating the interactions between three main factors (type of pollination, fruit set timing, and cultivar).

## Results

### Flower Morphological Sterility

The olive floral biology has been studied on four cultivars widespread in Southern Italy: Bella di Spagna, Coratina, Leccino, and Ogliarola barese. The olive orchards were localized in the provinces of Bari and Taranto, two environments characterized by different pedo-climatic features, thus preventing a combined statistical analysis. Cultivar identities of all plants selected for this study were previously confirmed by the use of SSR molecular markers and the comparison with standard cultivars (Alba et al., 2009; Baldoni et al., 2009). Plants belonging to the same cultivar have been confirmed to be clones without genetic variations (data not shown).

The fertility of each cultivar was investigated by the evaluation of NFI and numbers of perfect flowers (HF, flowers without malformations, and/or abortion of ovary or anthers) (Table 1). The analysis of variance revealed significant differences among the analyzed cultivars only in the Ionic site (West coast) with regard to the NFI, while the numbers of perfect flowers resulted highly significant in both environments.

**Table 1:** Analysis of variance of the mean number of flowers/inflorescence and the mean number of perfect flowers in the four olive cultivars evaluated at the Adriatic (BA) and Ionic (TA) orchards.

Source of variation	*df*	Adriatic coast		Ionic coast	
		NFI	HF	NFI	HF
Cultivars	3	7.06	2246.17***	27.70***	959.14***
Error	16	4.68	57.40	2.13	38.92


*df: degrees of freedom; NFI: number of flowers/inflorescence; HF, number of hermaphrodite flowers. ^∗∗∗^Significant differences at *p* ≤ 0.001.*

No significant differences were found for each cultivar analyzed in the two seasons in the same orchard (data not shown), but the Duncan’s test highlighted the presence of significant differences among the genotypes in the considered environments (Table 2). Due to the lack of significant differences in Adriatic orchard, the average values of flowers/inflorescence in each cultivar were evaluated in the Ionic orchard, where the mean value of this trait resulted 13.9. In particular, the cultivars Leccino and Ogliarola barese showed, respectively, the lowest (11.1) and the highest (16.8) average values, while the cv Coratina did not significantly differ from cultivars Bella di Spagna and Leccino, and cv Bella di Spagna did not significantly differ from cultivar Ogliarola.

**Table 2:** Comparison between the fertility mean values of each cultivar for two seasons and in both environments, by the application of Duncan’s test.

Cultivar	NFI		% HF
		Adriatic coast	
Bella di Spagna	9.1 ± 0.4		29.2 ± 1.7^B^
Coratina	11.1 ± 0.7		97.3 ± 0.4^A^
Leccino	11.7 ± 0.5		89.3 ± 1.4^A^
Ogliarola barese	11.4 ± 0.4		88.7 ± 2.1^A^
*Mean*	*10.8 ± 1.0*		*76.1 ± 31.5*
		Ionic coast	
Bella di Spagna	14.3 ± 1.2^AC^		59.9 ± 3.2^C^
Coratina	13.3 ± 0.7^AB^		92.1 ± 1.5^AB^
Leccino	11.1 ± 0.9^B^		98.2 ± 0.6^A^
Ogliarola barese	16.8 ± 0.3^C^		86.7 ± 2.4^B^
*Mean*	*13.9 ± 2.3*		*84.2 ± 16.8*


*Mean ±*SD*: data represent mean values of two seasons. Different letters within columns indicate significant differences among cultivars at *p* ≤ 0.01.*

With regard to the percentage of perfect flowers, the cv. Bella di Spagna was clearly distinguished from the other cultivars in both the orchards, always showing the lowest values (29.2 and 59.9). On the Ionic coast, the cultivars Leccino and Ogliarola barese were significantly different each other, but both similar to cv Coratina, while they did not show significant differences on the Adriatic site. Moreover, exclusively in the case of cultivars Bella di Spagna and Leccino, the production of perfect flowers was strongly and significantly influenced by environment as revealed by the Student’s *t*-test (29.2 versus 59.9 and 89.3 versus 98.2, respectively).

### Fruit Set

The fruit set percentage was evaluated in both free and self-pollination conditions at three time-points (40, 90, and 120 daf) as ratio between the fruit number and the number of previously detected flowers per tree. The time-points were chosen in relation to the premature fruit drop caused by physiological and environmental effects. A factorial analysis of variance has been carried out considering three main factors, i.e., type of pollination, fruit set timing and genotypes, and their interactions in a completely randomized design (Table 3). The variance of the three factors resulted highly significant in both environments (*p* ≤ 0.001), thus indicating that the fruit drops following the 40th day after blooming can cause substantial decrease in fruit number and thereby affect the olive productivity. Among the interactions, only “type of pollination” × “Genotypes” (P × G) resulted significant, the fruit set being a trait strongly dependent by the cultivar, and thus demonstrating the different behavior of each genotype with regard to open or controlled pollination. Moreover, since P × T and T × G were not significant, the effect of the “type of pollination” evidently does not change over the time and self-incompatibility can be indiscriminately evaluated at any of the considered time-points. Finally, the second order interaction (P × T × G) was not significant in both environments (Table 3).

**Table 3:** Analysis of variance of fruit set in the four olive cultivars evaluated in the Adriatic and Ionic orchards.

Source of variation	*df*	Fruit set	
		Adriatic coast	Ionic coast
Type of pollination (P)	1	1,058.68***	682.80***
Fruit set timing (T)	2	11.20***	32.79***
Genotype (G)	3	229.17***	80.57***
P × T	2	1.78	1.23
P × G	3	51.64***	58.30***
T × G	6	0.21	3.23
P × T × G	6	0.12	0.21
*Error*	*96*	*1*.*44*	*3*.*86*


*A complete randomized design was applied, considering the interactions between the main factors: type of pollination (P), fruit set timing (T), and genotypes (G). ^∗∗∗^ significant differences at p < 0.001.*

The behavior of each single genotype in terms of fruit set percentage was investigated in free and self-pollination conditions by the analysis of variance at the three time-points (40, 90, and 120 daf) in the two orchards, applying a complete randomized design (Table 4). In general, the variance resulted highly significant (*p* ≤ 0.001) throughout the three time-points on the Adriatic coast for both the pollination types. On the contrary, in the Ionic orchard, the variance resulted significant only in open pollination condition, with high level of probability at 90 and 120 daf (*p* ≤ 0.001) and lower level of probability at 40 daf (*p* ≤ 0.05). These results presumably reflect the strong influence of the Ionic environment throughout all the development period of self-pollination derived fruits and only during the first period of fruit formation in the case of free-pollination.

**Table 4:** Analysis of variance of fruit set percentages at 40, 90, and 120 daf in the four olive cultivars in free and self-pollination conditions for the two locations.

Source of variation	*df*	Fruit set (%) in free pollination					
		Adriatic coast			Ionic coast		
		40	90	120	40	90	120
Cultivars	3	73.23***	73.81***	75.09***	26.73*	46.62***	42.72***
Error	16	2.54	2.71	2.63	5.13	4.07	4.06

		**Fruit set (%) in self-pollination**					
		**Adriatic coast**			**Ionic coast**		
		**40**	**90**	**120**	**40**	**90**	**120**

Cultivars	3	10.51***	12.96***	12.35***	13.06	9.38	7.23
Error	16	0.33	0.44	0.39	3.83	3.19	2.91


*df degrees of freedom. ^∗^ and ^∗∗∗^ significant differences at *p* ≤ 0.05 and *p* ≤ 0.001, respectively.*

A minimal percentage of self-fertilization derived drupes was observed at all the time-points in both environments, thus indicating the partially self-fertile nature of all the analyzed cultivars, even if to a different extent. In particular, the highest self-pollination percentages were observed at the most relevant time point (40 daf) and in the orchard located on the Ionic coast (Table 5). The ratios between the fruit set percentage in self- and free-pollination conditions resulted constant throughout the time and equal to 1:8.5 and 1:3.5 in the Adriatic and Ionic orchards, respectively. This result highlighted the reduced influence of the type of pollination on fruit drops and thus justified the lack of significance in the P × T interaction (Table 3).

**Table 5:** Comparison between mean values of fruit set percentages of each cultivar at 40, 90, and 120 daf in both environments, in open- and self-pollination conditions.

Cultivar	Fruit set (%) in free pollination					
	Adriatic coast			Ionic coast		
	40	90	120	40	90	120
Bella di Spagna	0.54C	0.26B	0.21B	2.23b	1.01B	0.77B
Coratina	5.49A	4.29A	4.09A	5.33a	4.81A	4.18A
Leccino	3.01B	2.40A	2.26A	5.07a	4.05A	3.02A
Ogliarola barese	3.80AB	3.35A	3.32A	3.72a	3.43A	3.37A
*Mean*	*3*.*21*	*2*.*58*	*2*.*47*	*4*.*09*	*3*.*32*	*2*.*83*

	**Fruit set (%) in self-pollination**					
	**Adriatic coast**			**Ionic coast**		
	**40**	**90**	**120**	**40**	**90**	**120**

Bella di Spagna	0.08B	0.02C	0.02C	1.55	0.83	0.73
Coratina	0.68A	0.58A	0.53A	1.60	1.30	1.22
Leccino	0.19B	0.14B	0.13B	1.62	1.18	0.84
Ogliarola barese	0.59A	0.49A	0.48A	0.55	0.44	0.42
*Mean*	*0*.*39*	*0*.*31*	*0*.*29*	*1*.*33*	*0*.*94*	*0*.*80*


*Means without common letters indicate significant differences among cultivars at *p* ≤ 0.01 (uppercase letters) or at *p* ≤ 0.05 (lowercase letters). The Duncan’s test has been applied.*

In order to highlight which genotype contributed mostly and significantly to the fruit set variability, the comparison between the mean values for each single cultivar has been performed by Duncan’s test (Table 5). When left free to pollinate on the Adriatic coast, the cultivars Bella di Spagna, Coratina, and Leccino were clearly distinguishable, in particular during the first period of fruit development (40 daf). On the contrary, in the following stages, only the cultivar Bella di Spagna showed significant differences in fruit set in both the orchards. Similar results have been obtained during the medium-late development of self-pollinated fruits (90 and 120 daf), while at the early stage (40 daf), the cultivars Coratina and Ogliarola barese and the cultivars Leccino and Bella di Spagna showed similar behaviors in pair. Generally, the fruit set following self-pollination was significantly lower than that from open pollination.

The data of fruit set percentages in open- and self-pollination conditions were also analyzed separately for each cultivar at 40, 90, and 120 daf in both sites and showed that environment can significantly affect the degree of self-fertility (Table 5). Although all cultivars always resulted scarcely self-fertile, some differences have been recorded in the two environments. On the Adriatic coast, most of the cultivars resulted mostly self-incompatible, with very low ISI values in particular for cv Leccino (0.059), while the Ionic environment resulted in clearly higher ISI values (up to 0.821) and thus in a partial self-incompatibility of cultivars (complete self-compatibility only in case of the cultivar Bella di Spagna) (Table 6).

**Table 6:** Evaluation of the self-incompatibility index (ISI), i.e., the fruit set ratio in self- and cross-pollination conditions, in the four olive cultivars studied at 40, 90, and 120 daf both on Adriatic and Ionic orchards.

Cultivar	ISI value							
	Adriatic coast				Ionic coast			
	40	90	120	*Mean*	40	90	120	*Mean*
Bella di Spagna	0.148	0.076	0.095	*0.106*	0.695	0.821	0.948	*0.821*
Coratina	0.123	0.135	0.129	*0.129*	0.300	0.270	0.291	*0.287*
Leccino	0.063	0.058	0.057	*0.059*	0.319	0.291	0.278	*0.296*
Ogliarola barese	0.155	0.146	0.144	*0.148*	0.147	0.128	0.124	*0.133*


*Genotypes are classified as completely self-incompatible (ISI = 0), mostly self-incompatible (ISI = 0–0.15), partially self-incompatible (ISI = 0.15–0.3), and self-compatible (ISI = 0.3–1) (Androulakis and Loupassaki, 1990).*

### Analysis of *F*_1_ Progenies by Controlled Pollinations

The success of olive breeding crosses is strongly determined by the inter-compatibility of cultivars, that can significantly influence the rate of pollen contamination and determine the origins of progeny, actually fertilized or not by the expected pollen donor. In this study, the inter-compatibility among Coratina, as host cultivar, and three cultivars widespread in Southern Italy, including Leccino, Picholine, and Oliastro, used as pollen donors, have been exclusively assessed in the orchard on the Ionic coast due to the greater availability of trees per cultivar in this location and to the absence of nearby olive grove. All the chosen cultivars showed very interesting traits for breeding purposes, such as productivity, oil composition, and resistance to biotic and abiotic stresses.

The paternity ascertainment of the obtained offspring allows to evaluate the success of the performed pollinations. The SSR markers have been applied on DNAs extracted by seed endosperms for a fast discrimination among self- and cross-derived offspring, thus avoiding to dissect embryos or to stress the *in vitro* growing seedlings.

**FIGURE 1:**
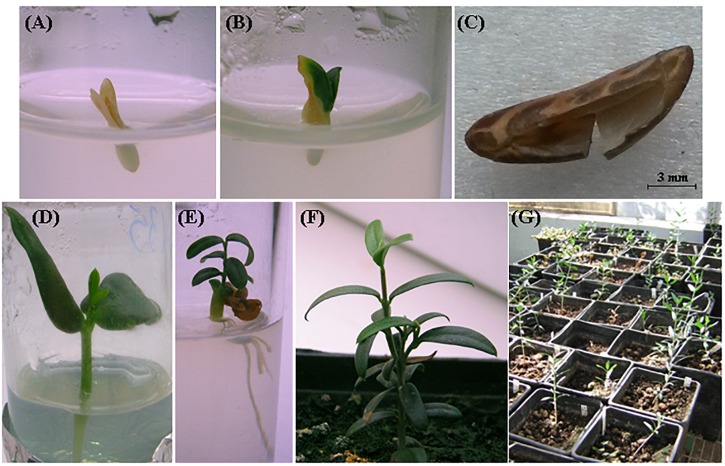
*In vitro* culture and development of olive mature embryos derived from controlled crosses. **(A)** Necked embryo 24 h after dissection; **(B)** changing color embryo after 3 days of *in vitro* cultivation; **(C)** collected endosperm; **(D,E)** olive seedling with young leaves and secondary roots; **(F,G)** plantlets moved to soil and greenhouse.

In Table 7, the numbers of harvested seeds, *in vitro* cultured mature embryos, and the final amount of greenhouse-acclimated plantlets are reported for each cross. Complete and healthy embryos were excised, on average, from the 62% of harvested seeds, since a certain percentage of empty seeds or embryos with structural anomalies were found for each cross. Most of the cultured embryos were able to successfully germinate and develop seedlings in less than 30 days with a quite high germination percentage, even if no growth regulators were added to the media (Figure 1). Unlikely, some embryos needed a prolonged *in vitro* culture period, thus clearly increasing the risk of microbial contamination; moreover, many plantlets suffered the transfer to the greenhouse and died during the acclimation phase. In total, 97, 118, and 100 healthy plants were obtained from C × L, C × O, and C × P crosses, respectively (Table 7).

**Table 7:** For each controlled crosses, the numbers of collected seeds, *in vitro* cultured embryos, recovered plantlets, and the number of identified self-derived progenies are shown.

Cross	Harvested seeds (n.)	*In vitro* cultured embryos (n.)	Plantlets (n.)	SSR marker	Alleles in parents (bp)	Self-derived progeny (%)
C × L	294	153	97	UDO-43	C: 174–198 L: 210–216	9.3
C × O	330	227	118	EMO-L	C: 198–198 O: 200–214	5.9
C × P	287	191	100	UDO-43	C: 174–198 P: 208–218	15.0


*The involved cultivars are listed as C = Coratina, L = Leccino, O = Oliastro, and P = Picholine.*

**FIGURE 2:**
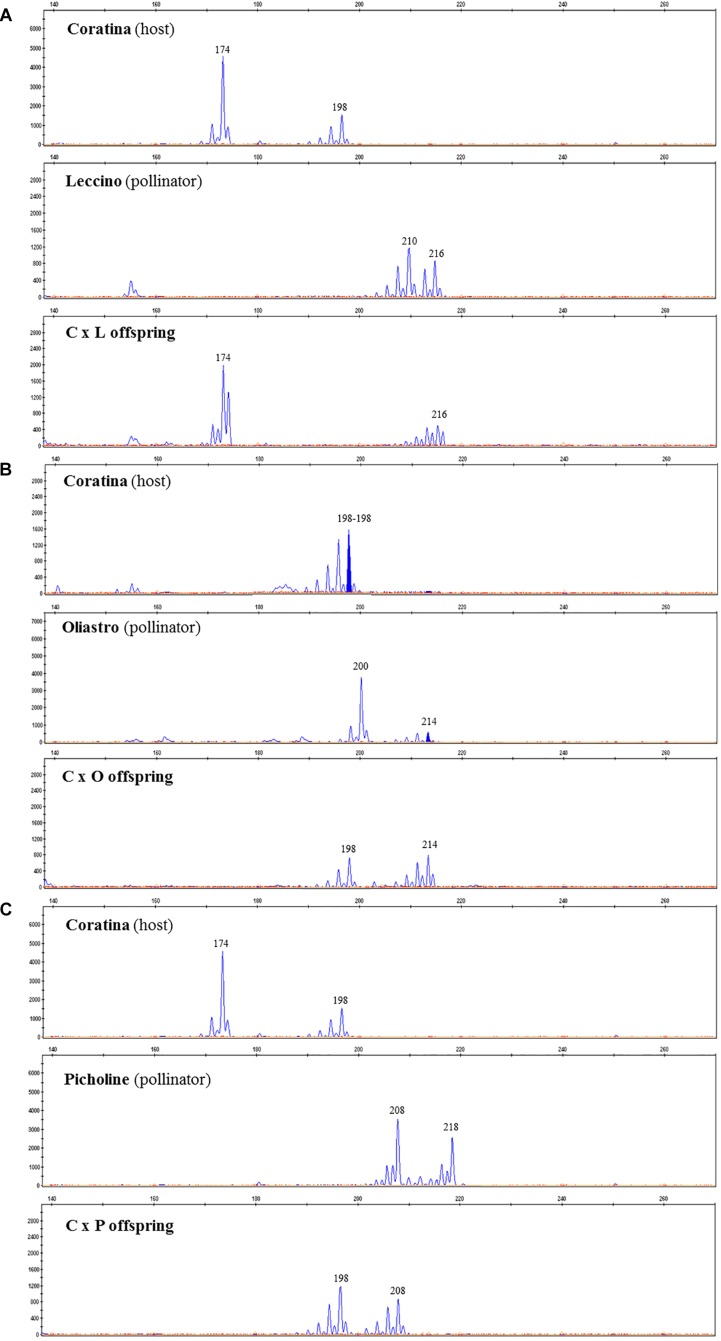
SSR electropherograms identified in C × L **(A)**, C × O **(B)**, and C × P **(C)** crosses. Each panel includes peaks (alleles) detected in the host (Coratina), the used pollinator (Leccino, Oliastro, or Picholine), and one of the correspondent recombinant progeny.

The progeny of each cross have been screened with two microsatellite markers, UDO-43 and EMO-L, chosen for their clear polymorphism between parents. The molecular profiles of parents are reported in Table 7 and some examples of the electropherograms are illustrated in Figure 2. Seeds having only maternal SSR alleles and thereby considered as self-pollination origin, have been found within each progeny. In these controlled crosses, the Coratina selfing incidence was rather low, ranging between 5.9 to 15.0%, thus highlighting its potential inter-compatibility with the used pollinators.

## Discussion

The knowledge of the reproductive biology in olive is a crucial issue for its improvement and the success of breeding programs. Most of the study on this issue mainly focus on the evaluation of self-compatibility for the most spread varieties and on the choice of the best pollinators, but data reported in literature are often contradictory (Bartolini and Guerriero, 1995; Ateyyeh et al., 2000; Cuevas et al., 2001; Lavee et al., 2002; Moutier, 2002; Collani et al., 2012; Farinelli et al., 2018).

The first part of our research examines the reproductive biology of four olive varieties (Bella di Spagna, Coratina, Leccino, and Ogliarola barese), widespread in the Mediterranean basin and highly promising for breeding purposes thanks to their physiological features and productivity (Muzzalupo, 2012; Rotundo et al., 2013; Perri et al., 2016). The fertility evaluation of these cultivars, estimated as NFI and as percentages of perfect flowers, has highlighted significant differences among the cultivars in both environments, particularly on the Ionic coast of Southern Italy, where the mean values of both parameters were constantly higher. In particular, the NFI in this orchard ranged between 11.1 and 16.8, resulting slightly lower than what reported by other authors who studied the same varieties in other environments (Guerin and Sedgley, 2007; Farinelli et al., 2015). On the other hand, on the Adriatic coast, where the mean values resulted even lower in comparison with those detected on the Ionic site, no significance emerged.

With regard to the percentages of perfect flowers, the differences among cultivars resulted significant in both environments, where in particular the Bella di Spagna cultivar considerably differed from the others, always showing the lowest values. This result is in line with what reported by Farinelli et al. (2015), in which this cultivar ranked in the category of genotypes with medium-low percentage of perfect flowers, while the majority of the analyzed genotypes generally clustered in the medium-high or high percentage of perfect flower groups. The other cultivars considered in our study, in particular cultivars Coratina and Leccino, have been confirmed to produce nearly all perfect flowers, as also observed by other authors (Guerin and Sedgley, 2007; Fayek et al., 2014; Farinelli et al., 2015). Additionally, the perfect flower percentage of olive cultivars analyzed in the two orchards and in different seasons might have been affected by environmental factors, such as temperature and water availability, thus causing a noticeable variation as in the case of Bella di Spagna and Leccino (Brooks, 1948; Hartmann and Panetsos, 1961; Seifi, 2009). Moreover, Rosati et al. (2011) suggested that olive pistil abortion (i.e., the percentage of staminate flowers) could be a fine maternal control of the available resources, causing competition among ovaries, and thereby the genetic differences in pistil abortion among olive cultivars may be explained by their different pistil mass and sink strength.

The fruit set evaluation is an essential trait in the study of the floral biology of a plant species, since it is related to productivity. In olive, only 1–2% of flowers present at the anthesis will usually give rise to fruits (Sibbett, 2005; Breton and Berville, 2013). In *sensu strictu*, this percentage may seem extremely low, but on the contrary, it guarantees a good productivity if considering the surplus of flower production per single plant necessary to guarantee an efficient wind- or pollinator-mediated pollination. The fruit set percentage following ovary fertilization is evaluated with certainty from the 40th day after bloom. For this reason, three time-points have been chosen for the analysis in our study: 40th, 90th, and 120^th^ daf. The investigation of three main factors, i.e., type of pollination, fruit set timing, and genotypes, by a factorial analysis of variance allowed to measure their single effect on plant productivity and underlined their high level of significance in both environments; clearly, a possible fruit drop following the 40th daf may seriously affect plant yield. Since the interactions “type of pollination” × “fruit set timing” and “fruit set timing” × “genotypes” did not show any significance, a certain reduction in fruit set may occur regardless the pollination conditions and, thereby, the evaluation of self-pollination incidence can be performed at any of the time-points. On the contrary, the interaction “type of pollination” × “genotypes” resulted significant, thus demonstrating how genotypes differently respond to self- and free-pollination, being the fruit set dependent from both these factors.

Values found in self-fertilization during all the time-points in both environments, take on a certain importance in the evaluation of the degree of self-incompatibility of the four cultivars, that might also explain the reduced influence of the type of pollination on fruit drops (no significance in “type of pollination” × “fruit set timing” interaction). In particular, the highest self-pollination percentage was found at the beginning of fruit formation (40th daf) in the orchard located on the Ionic coast.

The effect of all cultivars on fruit formation in free and self-pollination conditions was investigated by the analysis of variance in both environments. On the Adriatic coast, all cultivars clearly differentiated especially when free to pollinate; in particular, the first period of fruit formation was determinant to differentiate the cultivars Bella di Spagna, Coratina, and Leccino. The cultivar Bella di Spagna, whose olives are usually used for table consumption, always showed significant differences with the other cultivars, regardless the type of pollination, probably as a consequence of fruit size and of a mechanism that modulates the fruit set (i.e., mass of fruit) according to a negative relationship with ovary/fruit size (Rosati et al., 2010). This was particularly evident in the orchard in the Ionic coast, that is not surrounded by other olive groves and is typically characterized by higher temperature and absence of cold wind. This environment seems to have strongly influenced the entire period of plant productivity in self-pollination conditions, especially during the first 40 days. The pedo-climatic conditions of this orchard have generally allowed higher values of fruit formation in both pollination conditions. Thus, the different behavior of cultivars in terms of self-fertilization degree in the two environments highlights how the percentage of fruit set could be influenced by environmental and farming conditions. This may partially justify the conflicting results actually available in literature (Farinelli et al., 2006, 2018; Camposeo et al., 2012). For example, the cultivar Bella di Spagna has been first classified as self-sterile and successively as partially self-fertile from the same author (Ferrara et al., 1999), but the discrepancy of these data could be attributed to the different locations where experiments were performed. A similar situation has emerged for cultivar Coratina, one of the most widespread in Italian olive groves, that has been generally recognized as self-incompatible by some authors (Camposeo et al., 2012), partially self-compatible by others (Morettini et al., 1972; Lombardo, 2004), and totally self-compatible by others again (Del Gaudio, 1952; Godini, 2006). More recently, Fayek et al. (2014) have reported low average value of fruit set percentage for cultivar Coratina (0.23% in self-pollination test), thus classifying this cultivar as totally self-incompatible. Considering our data, three (Bella di Spagna, Coratina, and Leccino) out of four cultivars change the ISI classification between the two orchards, thus underling the environmental effect.

Thus, differences among cultivars and between locations in terms of fruit set and ISI values might be attributed to both physiological and environmental factors, but they are certainly indicative of an inter-varietal variability for which the genetic influence might play the major role. Variable self-fertility behavior depending on orchard and season was found in several fruit species, such as apple, peach, grape, almond, and cherry (Oberle and Goertzen, 1952; Godini, 1981; Szabó et al., 1996) as well as for the olive cultivars analyzed in this study. For example, it has been noticed that self-incompatibility breaks down following an increase in temperatures both in tree species, such as sweet cherry (Choi and Andersen, 2005) and olive (Al-Kasasbeh et al., 2005), and in some herbaceous species (Wilkins and Thorogood, 1992). In our research, low ISI values closer to zero were observed for most cultivars under the experimental conditions of the Adriatic coast, where the lowest value of self-incompatibility emerged for cultivar Leccino. This result could be correlated with the low free-pollination efficiency of this cultivar in this environment, when compared with free-pollination on the Ionic orchard. In fact, its ISI value considerably changed among sites, passing from 0.06 to 0.30, thus highlighting how the pedo-climatic differences among the two environments affect the fruit set of this cultivar, generally considered self-sterile (Farinelli et al., 2004; Spinardi and Bassi, 2012), and its level of self-compatibility. With regard to cultivar Coratina, for example, the influence of both environments in our study meant that this cultivar is mostly self-incompatible on the Adriatic coast and partially self-incompatible on the Ionic coast, according to the ISI values calculated as Androulakis and Loupassaki (1990). In general, it should be considered that pollen grains may suffer during the transport in the windy Adriatic environment, thus compromising the fertilization efficiency, as also suggested by other authors (Morettini and Pulselli, 1953; Ribeiro et al., 2003; Bonofiglio et al., 2010).

The knowledge about olive cross-compatibility relationships is a fundamental pre-requisite to reach the success of crosses in breeding programs and to design new orchards. The choice of cultivars in controlled crosses should base not only on some main desirable characteristics, such as productivity, resistance to stresses, olive/oil organoleptic properties, overlapping bloom times, self-compatibility, and so on, but also on the cross-compatibility relationships among cultivars. Thanks to its favorable characteristics, Coratina cultivar has been chosen as maternal line for our experimental crosses, in order to minimize the risk of self-pollen contamination in the progeny. Moreover, the crosses were exclusively performed in the orchard localized on the Ionic coast due to its pedo-climatic characteristics.

Cultivars producing high amount of viable and compatible pollen grains may potentially be ideal pollinators. Generally, the olive specie is characterized by high pollen production, a genetically and physiologically controlled trait, probably in relation to the need to cope with wind- or pollinator-mediated cross-pollination (Camposeo et al., 2012; Mazzeo et al., 2014). Obviously, pollen quantity is correlated with fruit production, that seems to be mostly independent of the amount of staminate flowers, while strongly determined by tree-environment interaction (Lavee, 1996). As potential pollinators of cultivar Coratina, three genotypes have been selected: Leccino, Picholine, and Oliastro. The cultivar Leccino has been proven to be an efficient pollen donor for many Italian and Croatian cultivars, despite its pollen exhibits low *in vitro* germinability (Selak et al., 2011).

Furthermore, the availability of a reliable and fast method to detect progeny actually fertilized by the expected pollen donor (the cultivar used as the father) is also desirable in a breeding program. Olive embryo isolation and *in vitro* culture has been widely applied by several authors in the years, especially for speeding up the seedling development (Rugini, 1984; Acebedo et al., 1997; Troncoso et al., 2003); on the contrary, the recovery and molecular analysis of the endosperms has not been reported so far. For example, the genetic assessment of *in vitro* germinated inter-subspecific hybrids derived from experimental crosses among cultivated olive and Asian and African wild relatives, has been reported by Cáceres et al. (2015), by AFLP and SSR analysis on DNA extracted from plants. Our application of SSR markers to DNA from endosperm rather than to DNA from seedling leaves is innovative for this purpose. Since olive endosperm is a reserve triploid tissue originated by the fecundation of the polar nuclei in the embryo sac, its analysis is equivalent to analyze embryonic tissue. The *in vitro* germination of excised embryos has been in depth demonstrated to rapidly break dormancy and shorten the time required to produce seedlings, speeding up olive breeding programs as well as rootstock production (Rugini et al., 1986; Acebedo et al., 1997; Troncoso et al., 2003; Germanà et al., 2014). In the present study, *in vitro* culture and endosperm analysis has been successfully used to initiate progeny evaluation. All cultivars used as pollinator seem to exhibit cross-compatibility with the female parent (Coratina), since pure progenies from all the controlled crosses were obtained. The number of empty seeds, as well as other characteristics such as seed weight or stone-less seed weight, can vary considerably among olive cultivars or progenies, thus partially explaining the differences in seed germination capacity and timing (Acebedo et al., 1997; Germanà et al., 2014). The cultivar Coratina has been reported to show a good seed germination percentage by Abdallatif et al. (2015). The plant growth rates per each progeny obtained in our experiment were quite high and the observed differences may be mainly due to their genetic background.

For this kind of analysis, the choice of SSR markers must base on the not-overlapping genetic fingerprints of the parents involved in the cross, that should possess at least one unique allele each. In our study, the use of two SSRs, i.e., UDO-43 and EMO-L, allowed to clearly discriminate all the genotypes used in the crosses. Endosperms with a SSR fingerprint matching exclusively with the maternal profile have been found within each progeny, even if with different percentages, and successively excluded from further analysis. The endosperm analysis by SSRs is a new strategy, here established for the first time, for a fast and easy selection of interesting plantlets, thus guaranteeing reliability, preserving embryo integrity, and saving time and costs.

## Conclusion

The multiple origins of domesticated *O. europaea* L. have led to a complex mating system that regulate sterility and compatibility, making it difficult to understand (Lavee et al., 2002). Despite of olive economic importance and the self-incompatibility occurrence among genotypes, the available information is still rather contradictory. Therefore, results from reproducible and integrated studies are very helpful to strengthen the information about olive compatibility, especially considering the strong influence of environment and climate on this trait. The results of our experiments may strongly contribute to shed light on the current debate about olive mating system, that at moment led to conflicting conclusions and are useful to make the right choice of the most appropriate cultivars by farmers when planning to set up new orchard or graft, and consequently the proper management of groves, in particular in Southern Italy. Moreover, the application of molecular markers to DNAs extracted by endosperms (and not from leaves) stored during the embryo rescue is a novelty in this type of analysis, since it allows to assess the hybrid nature of progenies from controlled crosses by the presence of male-specific alleles in the molecular patterns and to accurately accelerate their screening for olive breeding programs.

## Author Contributions

CM and WS conceived and designed the experiments. CM, GD, GB, and WS performed the experiments. WS analyzed and interpreted the data. CM contributed reagents, materials, and analysis tools. WS wrote and edited the manuscript.

## Conflict of Interest Statement

The authors declare that the research was conducted in the absence of any commercial or financial relationships that could be construed as a potential conflict of interest.

## Footnotes

1 www.olea-db.it

2 http://www.meteoam.it/
